# Recommendations for emergency departments receiving patients with vital signs absent from paramedics during COVID-19

**DOI:** 10.1017/cem.2020.389

**Published:** 2020-05-05

**Authors:** Brodie Nolan, Lucas B. Chartier, P. Richard Verbeek, Dirk Huyer, Laurie Mazurik

**Affiliations:** * Department of Emergency Medicine, St. Michael's Hospital, Toronto, ON; † Li Ka Shing Knowledge Institute, Toronto, ON; ‡ Division of Emergency Medicine, Department of Medicine, University of Toronto, Toronto, ON; § Department of Emergency Medicine, University Health Network, Toronto, ON; ¶ Toronto General Hospital Research Institute, Toronto, ON; # Toronto Central LHIN Lead for EM, Toronto, ON; ‖ Sunnybrook Centre for Prehospital Medicine, Sunnybrook Health Sciences Center, Toronto, ON; ** Office of the Chief Coroner for Ontario, Toronto, ON; †† Department of Emergency Medicine, Sunnybrook Health Sciences Center, Toronto, ON

**Keywords:** Cardiac arrest, coronavirus, emergency medicine, emergency medical services

## INTRODUCTION AND RATIONALE

Protection of frontline health care workers during the ongoing coronavirus disease 2019 (COVID-19) pandemic is essential. During the severe acute respiratory syndrome (SARS) outbreak in 2003, health care workers accounted for 21% of victims worldwide, and 43% of SARS patients in Toronto were health care workers.^1^ This was likely multifactorial, including a lack of personal protective equipment (PPE), unrecognized cases, and inadequate PPE used.^1,2^ This risk was greatest to nurses working in the emergency department (ED) and intensive care units.^2^ Aerosol generating medical procedures (AGMPs) are interventions that can cause airborne infectious particles to be propelled into the air. Due to their small size and potential to be suspended in the air for prolonged periods, additional precautions are required for health care workers who are exposed to AGMPs.

Obtaining an accurate history and COVID-19 risk factors for patients in cardiac arrest is difficult; therefore, we suggest presuming all patients presenting vital signs absent (VSA) to be infectious with COVID-19. The purpose of this study is to provide recommendations to enhance staff and patient safety during COVID-19 by reducing unnecessary exposure to AGMPs for ED staff, paramedics, and other ED patients when receiving patients with VSA. We suggest hospitals and emergency medical services engage with appropriate stakeholders and adjust accordingly for local practice.

## SUMMARY OF EVIDENCE

**Figure 1.**
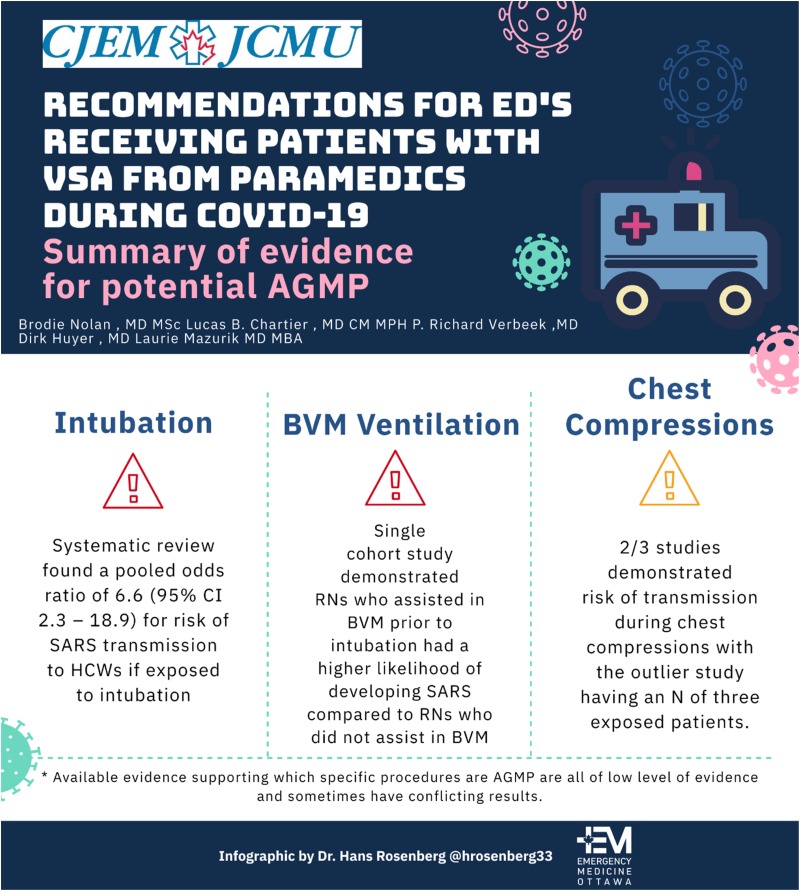
Summary of evidence for potential aerosol generating medical procedures.

Available evidence supporting which specific procedures are AGMP are all of low level of evidence and sometimes have conflicting results. Much of the data regarding the risk of aerosolization and transmission of pathogens causing acute respiratory infections comes from the SARS outbreak in 2003.^3^ Many of these studies were retrospective, making it challenging to draw conclusions from which specific procedures had risks of transmission.^3^ To aid in justification of our recommendations, we present a brief summary of available evidence regarding common AGMPs that occur during cardiopulmonary resuscitation (CPR) and the risks of transmission to health care workers (Figure 1).

### Intubation – Yes, evidence of increased risk of transmission to health care workers

A systematic review found a pooled odds ratio (OR) of 6.6 (95%confidence interval [CI], 2.3–18.9) for risk of SARS transmission to health care workers if exposed to intubation.^3^ Furthermore, Fowler et al. found that health care workers that had any involvement with intubation had a relative risk of developing SARS of 13.29 (95% CI, 2.99–59.04), despite all intubations being done in a negative pressure room with N95 mask, gown, gloves, and hairnets being donned by all health care workers (eye protection/face shields had variable use).^4^

### BVM ventilation – Yes, evidence of increased risk of transmission to health care workers

A single cohort study demonstrated nurses who assisted in bag-valve-mask (BVM) ventilation before intubation had a higher likelihood of developing SARS compared with nurses who did not assist with BVM before intubation (OR, 2.8; 95% CI, 1.3–6.4).^3,5^

### Chest compressions – Some, limited evidence of increased risk of transmission to health care workers

One case control study from China with 477 health care workers, of whom 15 of them did chest compressions demonstrated increased risk of transmission of SARS (OR, 4.5; 95% CI, 1.5–13.8).^6^ There are two cohort studies available with a pooled estimate of 1.4 (95% CI, 0.2–11.2).^3^ The first of these studies had a total of nine health care workers in Toronto who performed chest compressions on SARS patients, with one health care worker developing SARS (OR, 3.0; 95% CI, 0.4–24.5).^5^ The second study had a total of three nurses who performed CPR on SARS patients and none of them developed SARS (OR, 0.4; 95% CI, 0.01–7.8).^7^ In summary, two of three studies demonstrated risk of transmission during chest compressions with the outlier study having an N of three exposed patients. There is a lack of details of whether these health care workers were exposed purely to chest compressions or if airway management also occurred in this context, making the accuracy of determining AGMP risk of chest compressions difficult with the above evidence.

**Figure 2.**
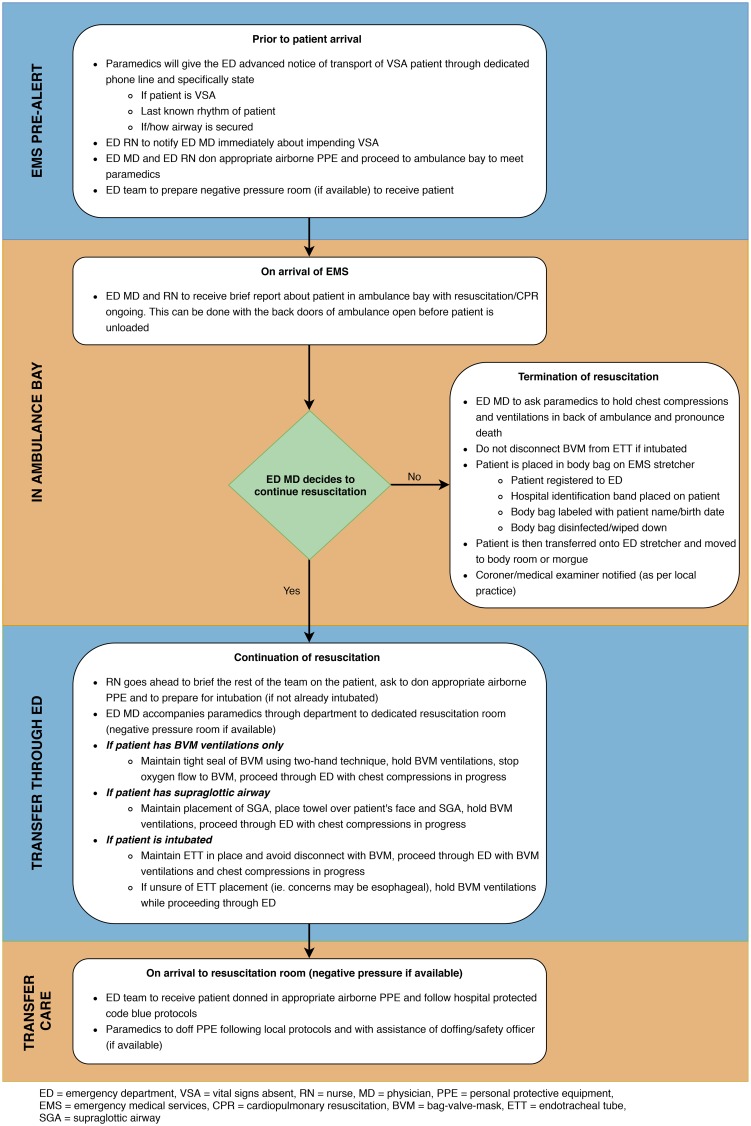
Recommended approach to receiving vital signs absent patients presenting to the emergency department with paramedics.

In SARS 2003, the outbreak was transmitted almost exclusively to health care workers in a health care setting, and it was not until near the end of the outbreak, that the means of transmission and measures to mitigate it were understood. The evidence gathered to support these recommendations was gathered in a time of crises and often retrospective. However, this does form a foundation to make these recommendations. Figure 2 is the recommended approach to receiving VSA patients presenting to the ED by paramedics.

## CONSIDERATIONS OF TERMINATION OF RESUSCITATION

Patients presenting to the ED in cardiac arrest in a nonshockable rhythm or asystole have a poor prognosis of being discharged neurologically intact.^8^ We believe that there may be cases where ED physicians could consider termination of resuscitation before the patient being brought into the ED. We recognize that pronouncement of death in an ambulance or ambulance bay is a considerable shift from normal practice for many emergency physicians. The justification of this procedure is to reduce the exposure of additional health care workers and other ill patients in the context of an expected resuscitation that is unlikely to be beneficial. Furthermore, it may reduce unnecessary PPE use in a time of PPE conservation by having only the ED physician donning airborne PPE.

Placement of the patient in a body bag in the ambulance or ambulance bay is potentially a significant way to contain the virus and reduce environmental contamination early, something that may be a silent contributor to transmission. Registration of the patient to the ED, along with appropriate identification and tagging of the body, will allow for medical documentation surrounding termination of resuscitation and ensure proper identification of the patient. Specifically, we suggest that a hospital identification band be placed on the patient's arm before the body bag is closed in the ambulance bay, the bag should be clearly labeled with a sharpie with the name and date of birth and the bag should be disinfected or wiped down with anti-viral wipes or a bleach solution before transfer into the hospital.

## AGMP RISK FOR PARAMEDICS

If there is an expected delay of more than 5 minutes for the ED physician to meet paramedics in the ambulance bay, paramedics should proceed to the identified resuscitation room with guidance from the ED nurses. Ambulances are small enclosed spaces without negative pressure capabilities and prolonged AGMP exposure puts paramedics at unnecessary risk.

## ADDITIONAL CONSIDERATIONS ON TRANSFER THROUGH THE ED

Many emergency medical services (EMS) are carrying oxygen delivery devices that are equipped with viral filters. We suggest maintaining and preserving these throughout the resuscitation. If one is not present on EMS arrival, providers could consider applying one before entering the ED.

Our recommendations are to continue chest compressions through the ED. This is a balance between the need of continuing high-quality chest compressions for patients in cardiac arrest and reducing environmental spread and potential contamination of the ED hallways. By deciding a patient should continue to be resuscitated into the ED, a physician has made the decision that this patient may have a chance of a good neurological outcome. We believe that, although chest compressions are likely to generate aerosols, the benefit to the patient of having continuous chest compressions outweigh the risks to hospital staff if our suggestions are followed. Hospitals could also consider draping a towel over the patient's face while being transferred through the ED.

If the decision to proceed with resuscitation is made, it will take some time for the other hospital health care workers to don their PPE. It should be understood that paramedics will be asked to continue CPR in the resuscitation room while this occurs with the assistance of the ED physician.

## THE NEED FOR A SYSTEMATIC APPROACH

Local and regional base hospital physicians who can advise paramedics on termination of resuscitation in the field or en route to hospital should review the indications for doing do. Hospitals and EMS should work together to reduce unnecessary delays and ensure that the dispatch center notifies the hospital of any inbound VSA or pre-arrest patients with as much notice as possible. It is possible that termination of resuscitation may have taken place during transport to the ED based on a patch to a base hospital physician so by the time the ambulance arrives paramedics are no longer doing resuscitation. In this case, the ED physician may still be required to complete the medical certificate of death as legally required.

Pandemics can overwhelm the capacity of a region to provide both critical and basic health care. In this setting, to preserve resources to provide care for those most likely to survive, advance discussions as a regional health network are needed to consider the threshold or indications to withhold resuscitation for VSA patients and have premade directives that can be activated to support this. Development of a protocol and an agreement with local paramedic services should be worked out before any practice is put in place.

This study provides recommendations to enhance staff and patient safety during COVID-19 while trying to balance a patient's need for high-quality CPR during cardiac arrest. These recommendations are meant to serve as a framework and will require adaptation for local practice.
